# Dental Neuralgia

**Published:** 1870-09

**Authors:** S. P. Cutler


### ARTICLE III.

### *Dental Neuralgia.*

By S. P. Cutler, M.D., D.D.S.

**Read before the Tennessee State Dental Association.**

Dental Neuralgia. *Surgical Operation.*—An Irish woman about 35 years old who had been the victim of neuralgia in the left inferior maxilla of the most intense character, in consequence submitted to surgical interference for relief, in the hospital at Cleaveland, Ohio, about two years before the time of the second operation, which I witnessed at the hospital at Cincinnati, in the winter of 1867.

The first operation consisted in trephining the lower maxilla with a small trephine opposite the sapient tooth (which had been previously extracted, also all the teeth on that side to the cuspid without relief,) by making a longitudinal incision just at the lower border of the jaw, dissecting up the flap and cutting through the masseter to the bone, then sawing down to the dental canal, and with the trephine removing a small section of the inferior dental nerve as well as the vessels.

This operation afforded only temporary relief, the paroxisms returniug with the most intense suffering at stated periods, showing conclusively that the diagnosis had been at fault, and the operation an entire, failure as might reasonably have been expected after the removal of said teeth failed to afford relief.

The second operation was performed by Prof. Blackman, of the Ohio Medical College, Cincinnati, during the winter of 1867-'68, at the Charity Hospital, before the medical and dental faculties and classes.

The poor woman at the time was about seven months enciente. She was placed on the operating table in the amphitheatre and put under the influence of chlorform, after the Professor had made an interesting statement concerning the operation. The operation was commenced by making a longitudinal incision first under the lower margin of the bone some two inches in length, dissecting up the flaps and making sufficient room for the operation. The facial artery being ligated a second incision was made down to the bone about two inches in length, extending a little in front of the mental foramen, then cutting down to the maxillary nerve canal with the small trephine, about opposite the last molar region; then with a bone excising forceps the bone was removed in front to the mental foramen, cleaning out the entire canal and foramen, which completed the operation, the integuments being saturated and dressed in the usual way. After the woman came to, she said she felt perfectly relieved and that she had rather undergo the operation every week than to suffer what she had.

Prof. Blackman stated that he did not have much confidence in the final result of the operation, and that if there was a return of the disease he intended to make an operation through the antrum with the small trephine and remove Meckle's ganglion.

The disease having returned in the course of a few days after the first operation as Prof. B. anticipated, he performed the operation as above contemplated with entire relief, and no symptoms of return up to the time I saw him last which was two weeks after his first operation. Whether the success was final or not I never heard.

The patient, a laboring woman, could assign no cause for the disease, only cold from exposure, her teeth being sound. In the first place, it was mal-practice to extract the teeth on that side, when there was no indication of disease about them. In the second place, it was doubtful practice to trephine the bone and remove a section of the inferior dental nerve, unless the symptoms were clear and pointed. In the third place, it was not only doubtful surgery but clearly malpractice to trephine the second time as after the removal of so much bone and the nerve, provided the first operation had been well performed and the section of nerve entirely removed, there could not have been any reunion or reestablishment of nerve; neither is there any anastomosis that could possibly communicate with the brain anterior to the point of the first trephining, a fact which should have been known to the Surgeon, and negatived the second operation altogether. All the operations performed on this poor woman were but so many gropings in the dark, without any guarantee at all of final relief. If the cause of the disease was not removed, a return of it was certain to follow sooner or later, perhaps with renewed energy, the operations serving only the purpose of temporary counter irritation. In this obscure case the cause may have existed still back of the points reached by the respective operations.

In the first place our knowledge of the exact pathology of neuralgia is very obscure, owing mainly to the fact of our limited knowledge of mere force or function, whatever it may be, for, when in a normal condition, effects follow cause in harmonious sequence, just as much as the falling of an apple to the earth responds to a cause called gravity, one being physiological, the other cosmical, and both under one common head denominated physical.

Normal physiological causes produce normal effects, and abnormal causes abnormal effects, pain and neuralgia being the result. Here then there must be looked for a violation of law, and neuralgia the result, as all violations of law have defined fixed penalties being natural sequences.

There may be, in some instances, spicula of bone growing in upon the trunk of a sentient nerve, producing disturbance between that point and the brain, causing pain from irregular neural transmission of force to the brain. Or there may be congestion in the neurilemma and thickening, thereby causing pain from pressure, or there may be strictures of the trunk resulting from external inflammation either acute or subacute, producing pressure on the axis cylinder and pain, or the heat of inflammation alone may be sufficient to cause pain by a preponderence of the chemical over the vital forces, oxidation subsidising nutrition which causes disturbance in the normal forces, nerve force being converted into heat force to an abnormal degree. Any traumatic injury of a nerve may heal by the first intention, without any neuralgia, or take on a morbid healing process producing either temporary or permanent neuralgia. The fangs of the lower wisdom teeth when long and the depth of jaw shallow, may in their growth intrude upon the maxillary canal and produce neuralgia by pressure on the trunk, which can readily be diagnosed and relief always obtained by extraction, if not by any other means. Tooth-ache from any cause whatever, is nothing less than dental neuralgia effecting the entire nervous system by sympathetic relation through ganglionic media, they being the outposts of the great central sensorial reservoir, where all dispatches are received on the one hand, and distributed on the other, to every point in the organism.

Dental or tooth neuralgia is more radically diagnosed and accounted for than any form of neuralgia, from the fact that the causes are readily arrived at, and acceptible when existing as such, uncomplicated with any other pathological condition. In the first place the dental pulp is enclosed or surrounded by solid unyielding eburnia substance, or ivory walls incapable, as is the case with sarcode, of diluting or yielding to the expansive force of over action of the heart and arteries, as in inflammations causing pain and intensified according to degree of heart's action the result of pressure, and thereby obstruction of nerve transmission, then again independent of pressure, preternatural chemical action or oxidation and corresponding diminution of nutrition, causing irregular or abnormal transmission of neural force resulting in pain ; or there may be a still lower degree of pressure, the result of stacis of blood or stagnation of actual inflammation, causing only promonitory symptoms of what is about to follow if not relieved .There is another condition called hyperæsthesia or exalted sensitiveness of dentine, the cause of which is exceedingly obscure, not depending on any of the above named conditions, which is annoying to both dentist and patient in operating on the teeth,—a condition of things demanding of our hands more scientific knowledge than we at present possess. Alveolar abscess, caused by dead teeth and fangs, is another common cause of dental neuralgia, which need seldom ever be incorrectly diagnosed, and the remedy always reliable, that is removal of such teeth and fangs if not otherwise curable.

Dead and diseased teeth and fangs cause neuralgia by their disturbance acting more or less as a foreign body in the jaw bone, the disturbance consisting of sub-acute or chronic inflammation of the bone extending to the trunk of the dental nerve and to the brain. Increase of blood produces pressure and undue oxidation in the vessels, causing irregular nerve transmission, resulting in painful impressions in the cerebrum. Relieve this trouble, the inflammation must be subdued, and the circulation of the blood restored to its normal condition, which is always more difficult to do in a bony structure than in any other tissue, recovery to perfect health in said tissue requiring more time than in any other, though when perfectly restored the tissue is perfectly normal bearing no scar. There can be no disease in a part where the circulation of blood is perfectly normal, there must first always be an over or an under action of the capillaries in any tissue before disease is established, even when resulting from traumatic injuries, cause and effect being always inseperable companions in the first instance. Why pain follows local disturbance is one of the life mysteries not yet fully comprehended; or why man and animals are sentient creatures at all is equally mysterious and incomprehensible.

To cure neuralgia, or in fact any other disease, the first step should be to try and find the cause, and if practicable remove such cause as a preliminary step, then the effect may in many instances subside without any farther interference—as an instance, the extraction of diseased teeth generally affords perfect and permanent relief without any further interference.

---

